# EO-1 data quality and sensor stability with changing orbital precession at the end of a 16 year mission

**DOI:** 10.3390/rs9050412

**Published:** 2017-04-27

**Authors:** Shannon Franks, Christopher S.R. Neigh, Petya K. Campbell, Guoqing Sun, Tian Yao, Qingyuan Zhang, Karl F. Huemmrich, Elizabeth M. Middleton, Stephen G. Ungar, Stuart W. Frye

**Affiliations:** 1 NASA’s Goddard Space Flight Center, Code 618, Greenbelt, MD 20771, USA; 2 Stinger Ghaffarian Technologies (SGT), Greenbelt, MD 20770, USA; 3 University of Maryland, Baltimore County, Baltimore, MD 21250, USA; 4 University of Maryland, College Park, College Park, MD 20740, USA; 5 Universities Space Research Association, Baltimore County, Columbia, MD 21044, USA

**Keywords:** EO-1, Hyperion, Advanced Land Imager, ALI, Precession, Solar Zenith Angle, data quality, Landsat-7

## Abstract

The Earth Observing One (EO-1) satellite has completed 16 years of Earth observations in early 2017. What started as a technology mission to test various new advancements turned into a science and application mission that extended many years beyond the satellite’s planned life expectancy. EO-1’s primary instruments are spectral imagers: Hyperion, the only civilian full spectrum spectrometer (430–2400 nm) in orbit; and the Advanced Land Imager (ALI), the prototype for Landsat-8’s pushbroom imaging technology. Both Hyperion and ALI instruments have continued to perform well, but in February 2011 the satellite ran out of the fuel necessary to maintain orbit, which initiated a change in precession rate that led to increasingly earlier equatorial crossing times during its last five years. The change from EO-1’s original orbit, when it was formation flying with Landsat-7 at a 10:01am equatorial overpass time, to earlier overpass times results in image acquisitions with increasing solar zenith angles (SZAs). In this study, we take several approaches to characterize data quality as SZAs increased. Our results show that for both EO-1 sensors, atmospherically corrected reflectance products are within 5 to 10% of mean pre-drift products. No marked trend in decreasing quality in ALI or Hyperion is apparent through 2016, and these data remain a high quality resource through the end of the mission.

## 1. Introduction

High quality data are of utmost importance for scientific studies and measurement stability over time is critical to evaluate seasonal, annual, and decadal changes [1]. In satellite remote sensing, analysts need to be sure that the imagery comes from well calibrated and characterized sources and the EO-1 satellite is no exception.

EO-1 was launched on November 21, 2000 as a one-year technology validation and demonstration mission. EO-1, at 572 kg total mass, approaches the smallest (< 500 kg) class of spacecraft. It was initially tasked with testing advancements that could potentially increase sensor performance while reducing instrument mass, power consumption, and cost. The three primary instruments on the EO-1 spacecraft were designed to acquire visible through near-infrared (VNIR) and shortwave infrared (SWIR) wavelength information in the solar reflected spectrum [2]. These are the Advanced Land Imager (ALI), the Hyperion, and the Linear Etalon Imaging Spectrometer Array (LEISA) Atmospheric Corrector (LAC), which is not discussed further in this contribution.

EO-1 demonstrated that its 8-band multispectral (MS) imager, ALI, provides a significant improvement over the Landsat 7 Enhanced Thematic Mapper plus (ETM+) and previous Landsat Thematic Mapper (TM) instruments due to increased signal to noise ratio, while decreasing instrument size and weight. EO-1 also validated the scientific value of orbital imaging spectroscopy with Hyperion, the first spaceborne hyperspectral land imaging instrument. EO-1 also demonstrated that a moderate-spatial/high-spectral resolution imager could be used in self-correction of atmospheric effects to retrieve the apparent surface reflectance from top of atmosphere (TOA) radiances, and systematic errors [3]. It was originally thought that these new technologies could improve the TM/ETM+ sensor series found on the Landsat 4 – 7 satellites [4,5] and indeed, some of these technologies were utilized for the Landsat 8 Operational Land Imager (OLI) sensor launched in 2013, with, for example, the adoption of the push-broom style sensor and improved quantization [6,7].

With no indication of sensor degradation through the end of the one-year baseline mission in 2001, the EO-1 mission was extended, allowing ALI and Hyperion to continue collecting images [3]. The mission was chartered to collect and distribute ALI MS and Hyperion hyperspectral products in response to Data Acquisition Requests (DARs), which were soon established as a collaboration with the U.S. Geological Survey (USGS) Earth Resources Observation and Science (EROS) Data Center (EDC). There were four extensions of the mission [8], during which normal operations continued and the daytime imagery were used in numerous science investigations [9] ranging from characterizing forest structure [10–12], water quality [13,15] and desert dust flows [16], to disaster monitoring activities [17–19]. Additionally, the nighttime imagery was greatly utilized by the volcano monitoring network [20,21].

### 1.1. Orbital Precession Rate Change

**Figure 1:**
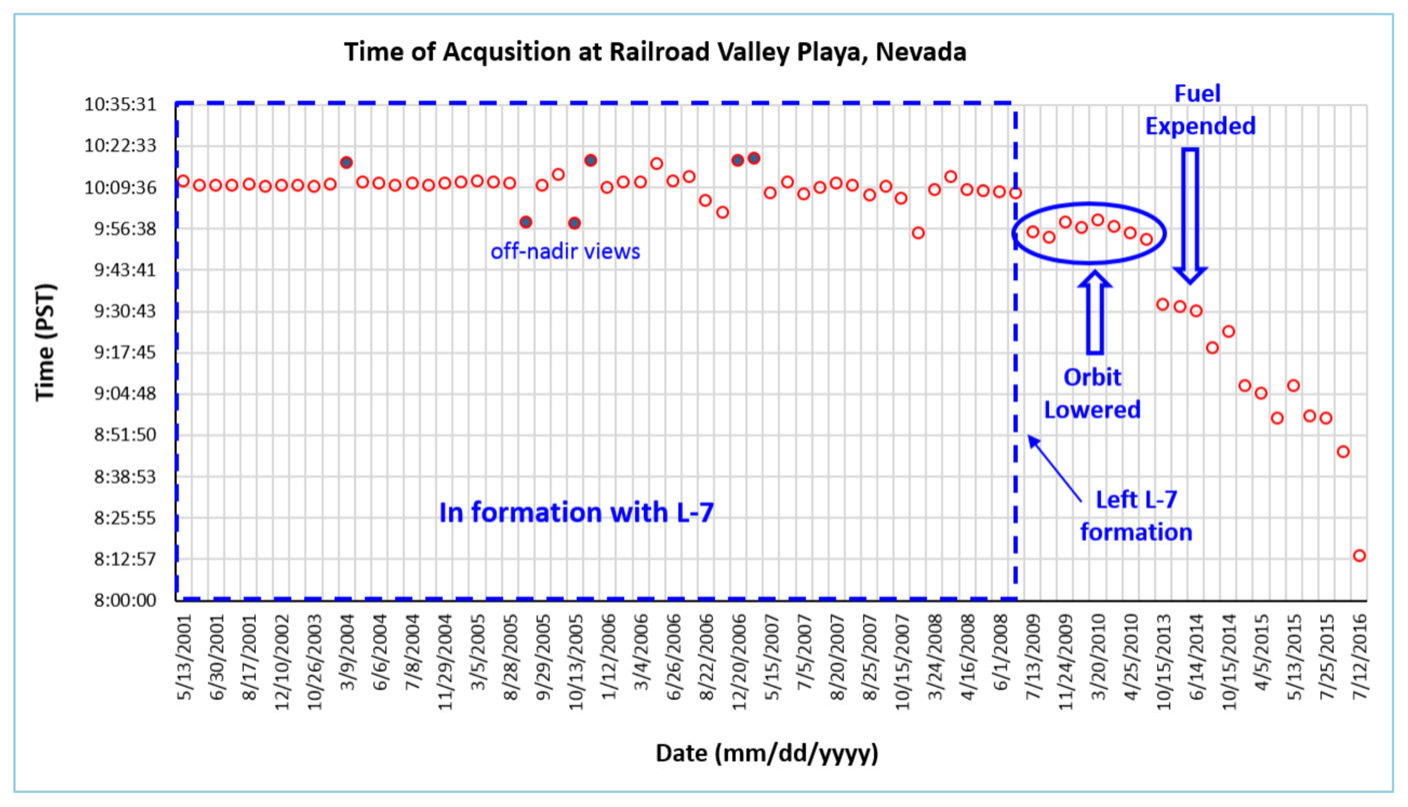
EO-1 changes in precession rate started in 2011. Acquisition time at the Railroad Valley Playa declined from 10:12 to 8:14, reaching 8 AM in October, 2016. The stability of the overpass time from 2001 to 2008 is shown (within dotted box), for nadir views (open circles) as well as off-nadir views (filled circles). A 15 minute change in overpass time is indicated within ellipse, after EO-1 left the Landsat-7 formation. The decline of overpass time is indicated by the downward arrow, after onboard fuel was expended.

From 2001 to 2007, EO-1 was flying in orbital formation with Landsat 7 (one minute behind). In late 2007 EO-1 began a de-orbit procedure, but barely a month into the process NASA gave EO-1 a re-entry waiver, and all remaining fuel was used to maintain the current orbit, which by then was slightly (~5 km) lower than Landsat 7. In 2011 EO-1 ran out of maneuvering fuel and the orbital degradation began, with a slowly changing rate of precession leading to increasingly earlier ground overpass times (Figure 1). A second waiver was issued for EO-1 to continue operations to study the effects of changes in precession on data quality.

**Figure 2:**
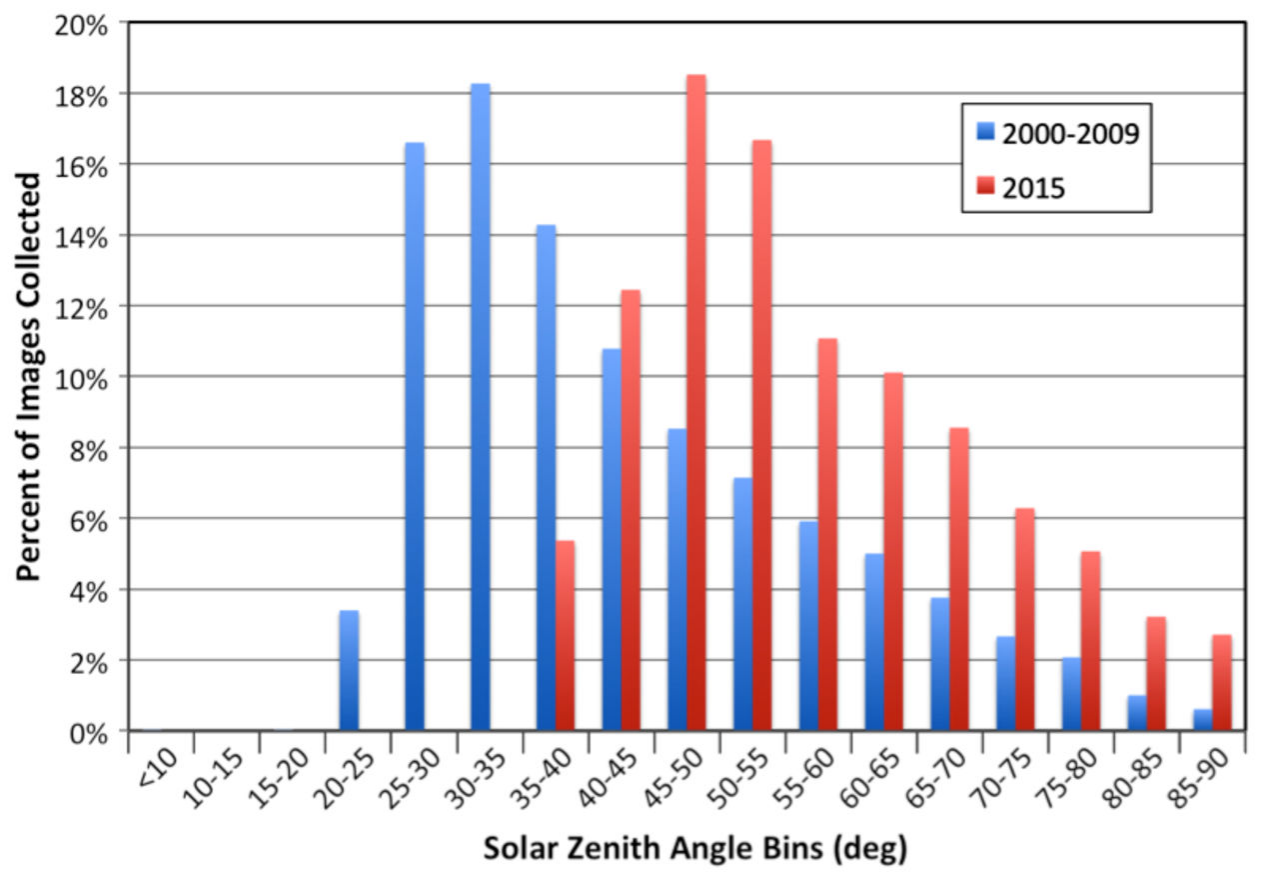
Solar zenith angles (SZAs), measured in degrees, of daytime Hyperion images. Due to the earlier acquisition times for the imagery, the distribution of SZAs at overpass times during 2015 shifted to larger values (red bars) than during the earlier normal mission operations (blue bars).

In 2011, EO-1 lost its ability to maintain an exact sun synchronous precession orbit when the satellite depleted its onboard maneuvering fuel and could no longer make corrective “inclination burns”. EO-1’s orbital plane is still precessing, but not at the rate required to achieve precise Sun synchronicity, resulting in nadir observation times drifting towards earlier local overpass times. The implication for this is that specific targets are now viewed under different illumination conditions per month of the year, as compared to earlier phases of the mission (Figure 2).

**Figure 3:**
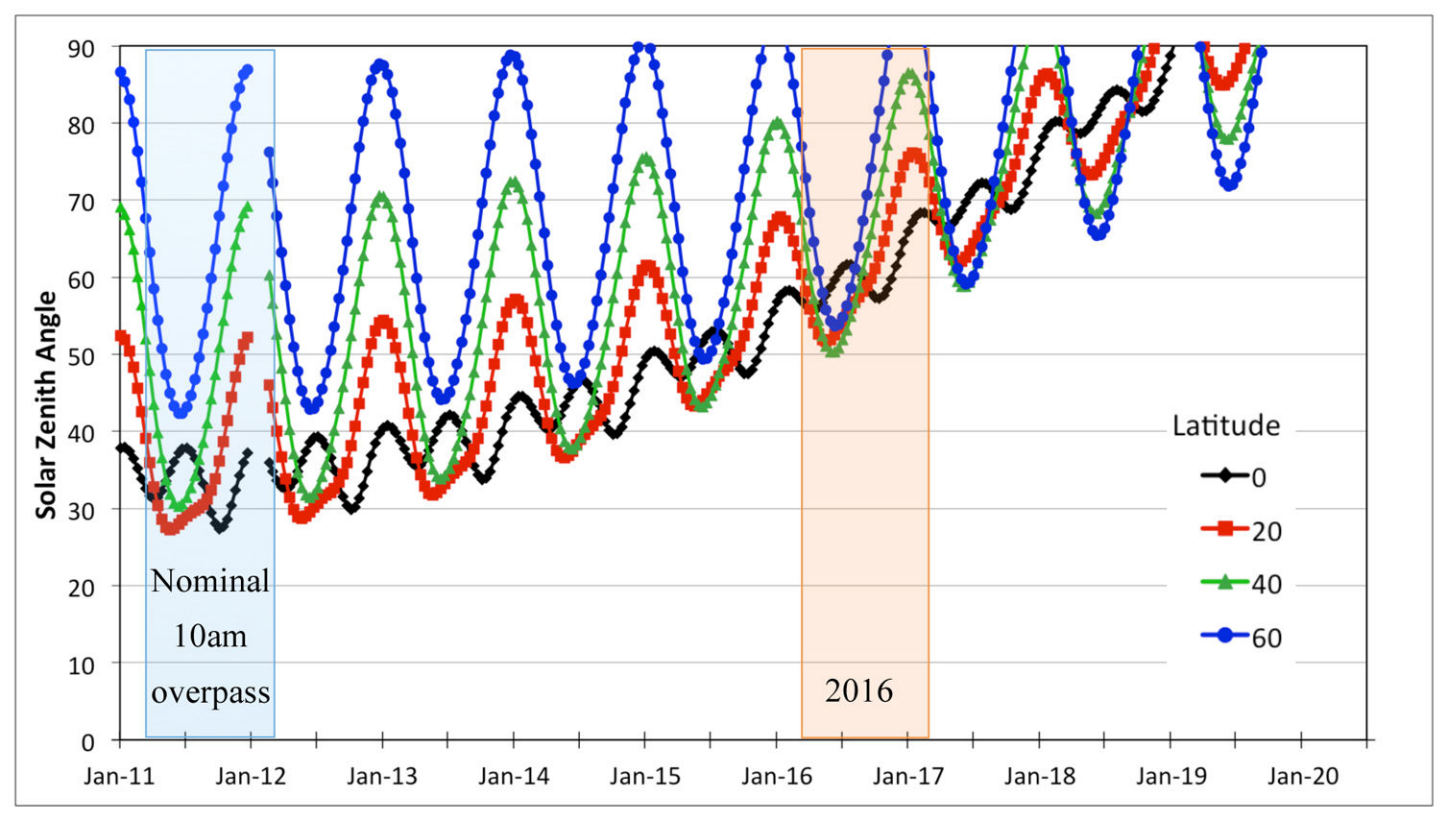
Solar Zenith Angles (SZAs) at EO-1 overpass time showing the effect of changing seasons and latitudes on SZAs as overpass time drifts to earlier times (colored lines, the colors indicating the latitude of the observation as shown in the legend). The blue shaded region shows SZAs at various latitudes during nominal operations, compared to SZAs experienced in 2016 (orange shaded region).

Solar zenith angle (SZA) depends on local overpass time, latitude, and date. The earlier overpass times experienced in the last few years of the mission result in larger SZAs. However, these values of SZAs have already been experienced by EO-1 for imagery collected at different latitudes and seasons. As can be seen in Figure 3, approximately 60% of the time in 2016 the SZAs at EO-1 overpass times were within the previously experienced range of SZAs for that latitude.

There are several potential impacts of changing orbital precession (also referred to as “drift” or “decay”) [22]:

1. Larger SZAs due to the sun being closer to the horizon may reduce the quality of the signal by having weaker irradiances and a longer atmospheric path for radiances to traverse, which decreases the signal to noise (SNR) ratio of the data and complicates atmospheric correction procedures.
2. There is a change in the number of instances of cloudy data, which might be expected to increase in temperate zones as early morning haze is more often present and to decrease in tropical zones where convective clouds dominate [23].
3. The bi-directional reflectance distribution function (BRDF) of the data changes as the influence of shadows increases in concert with the illumination angles, and somewhat larger footprints are viewed. [24]

Understanding these effects of changing overpass times helps future mission planners to evaluate overpass times for sun synchronous missions as well as understanding the issues involved with integrating data from multiple satellites with different overpass times as proposed in future actual and virtual constellations. EO-1 became a natural experiment in this problem providing unique opportunities to evaluate these questions due to its long time series to which comparisons can be made.

Here, we examine quality and stability of the daytime imagery acquired by two EO-1 sensors to determine the effect of orbital precession on data products with changing SZAs and footprints in areas with different surface characteristics. We used multiple approaches in several locations with varying surface properties to highlight different aspects of the effects of EO-1’s changing precession over time. We selected three vegetation sites and two Committee on Earth Observing Satellites (CEOS) Pseudo Invariant Calibration Sites (PICS) to evaluate data quality and stability changes.

## 2. Data and Methods

### 2.1 EO-1 Instrument Characteristics

The EO-1 ALI is an 8-band MS imager having a 15° Wide Field Telescope (WFT) and a partially populated focal plane occupying 1/5th of the field-of-view, giving a ground swath width of 37 km. Hyperion is a grating imaging spectrometer with a 7.7 kilometer swath and it provides 220 functional bands with an approximately 10 nm sampling interval from 430–2400 nm [3]. Both ALI and Hyperion have 30 m nadir ground pixels to match Landsat-7. All collected data are archived and distributed by the USGS/EROS data center [25].

### 2.2 Study Methods

Five different study sites were selected to evaluate the impacts of precession on surface reflectance from Hyperion and ALI at different latitudes and land cover types to characterize sensor performance (Table 1). It would have been desirable to have more test sites in differing regions of the world, but it was difficult to find time series data, which was needed to show effects of changing precession over time. This was because EO-1 was never conceptualized to be a robust repeat sensor. For this reason, several of our selected sites were PIC sites.

1. Park Falls, Wisconsin: The Normalized Difference Vegetation Index (NDVI) [26,27] obtained from ALI TOA reflectance was compared with that from Landsat NDVI, and an NDVI difference was computed for a mixed forest site in multiple years;
2. Howland Forest, Maine: The NDVI obtained from Hyperion surface reflectance was compared to the NDVI obtained with the Moderate Resolution Imaging Spectroradiometer (MODIS) for an experimental mixed forest, in multiple years;
3. U.S. Department of Agriculture/Beltsville Agriculture Research Center (USDA/BARC) in Beltsville, Maryland: The temporal change in the Hyperion surface reflectance was evaluated using 1^st^ derivative analysis, in an agriculture site over multiple years;
4. Rail Road Valley Playa (RRVP), Nevada: For a Pseudo-Invariant Calibration Site (PICS), a Hyperion surface reflectance time-series was evaluated for a bright desert target site; and
5. The Libya-4 PICS, Libya: Statistical evaluation of the change in surface reflectance obtained in different spectral intervals and over time was evaluated using a dense Hyperion surface reflectance time-series for a bright desert target site.

#### 2.2.1. Park Falls Wisconsin – EO-1/ALI NDVI vs. Landsat NDVI

Park Falls, Wisconsin was chosen as the study site for this experiment because of the density of EO-1 imagery available and the diversity of included land cover types. This site falls within the Worldwide Reference System (WRS) path 25, row 28. One ALI image per year from summers of 2001 to 2016 were selected and a corresponding Landsat image was chosen to minimize the difference between the dates of acquisition (Table 2). For the twelve pairs of images, the cumulative delta days between all acquisitions were 19 days, averaging just 1.58 days/pair. Comparison of the spectral bandwidths (Table 3) of each sensor shows that all bands except the Near Infrared (NIR) have very similar wavelength ranges. Sun and view angle differences also play a part in NDVI calculation [28]. Solar zenith angle differences were minimalized as acquisitions of the comparitive scenes were all within a few days of one another so solar zenith angles were all very close. When considering view angles, Landsat images are always nadir viewing whereas EO-1 has the ability to view up to one neighboring WRS path on each side. With this in mind, the EO-1 images were selected to reduce impacts of view angle and all but one had view angles less than 7.5 degrees. The 2016 ALI image had an off-nadir angle of 20 degrees, but it has been demonstrated that NDVI is not highly effected until this angle is greater than 25 [28].

Data for the EO-1 and Landsat sensors were downloaded from USGS in units of TOA reflectance and had radiometric and systematic geometric corrections applied to achieve a Level One Terrain corrected and ortho-rectified (L1T) image product. An NDVI product was obtained from ALI and Landsat scenes, as a normalized difference spectral index using Equation (1) [27]:

(1) $$\mathrm{NDVI}=\frac{\rho_{\mathrm{NIR}}-\rho_{\mathrm{red}}}{\rho_{\mathrm{NIR}}+\rho_{\mathrm{red}}}$$

Each Landsat image was subset to the boundaries of the EO-1 ALI imagery to have exactly the same region of interest for statistical calculations. A data cube was built with these stacked images. To establish land cover classes, the 2011 National Land Cover Database (NLCD) [29] was downloaded and overlayed onto the data stacks.

For the analysis, the NDVI associated with each pixel was differenced (ALI minus Landsat) and those differences were averaged per NLCD land cover class. In our experience from visually looking at pixels with large NDVI differences, any delta NDVI between the ALI and Landsat data greater than 0.15 were most likely due to something other than sensor difference. These other differences could be from clouds, a rain event, or a land disturbance in one of the images, and we removed them from the comparison.

#### 2.2.2. Howland Forest, Maine – Hyperion NDVI comparison to MODIS NDVI

Three Aflux tower sites (US-Ho1: 45.20° N, −68.74° W; US-Ho2: 45.21° N, −68.74° W; and US-Ho3: 45.21° N, −68.73° W) in the Howland Experimental Forest, Maine were chosen for the analysis of Hyperion NDVI vs. MODIS NDVI, utilizing imagery collected between 2003 and 2014, with representation from all seasons. All three sites were mixed evergreen forest with a warm summer climate and significant precipitation in all seasons. The mean average temperatures are slightly over 5°C and the elevation ranged from 60 to 90m. The spatial resolution of nadir MODIS data is either 500 m or 1 km, while the spatial resolution of Hyperion data is 30 m. Thus, the reflectance values of Hyperion data have been aggregated spatially to match MODIS pixels at either 500 m or 1 km resolution. To see phenology variability using the EO-1/Hyperion reflectance data product, the Hyperion NDVI data were compared with NDVI derived from MODIS by differencing the spectral pixel values.

The Hyperion Level 1 GST (L1GST) product having radiometric and geometric corrections as well as a systematic terrain correction were downloaded and used for this analysis. The Hyperion L1GST images were atmospherically corrected using the Atmosphere Removal Algorithm (ATREM) [30] [31]. Hyperion NDVI data (using Eqn. 1) were calculated from the surface reflectance using bands 27 – 32 and 49 – 54 of the L1GST product. The NDVI data from the MODIS satellite instrument were calculated from surface reflectance in MODIS bands 1 (620 – 670 nm) and band 2 (841 – 876 nm). MODIS Level 1B calibrated radiance data (MOD021KM and MOD02HKM) and geolocation data (MOD03) were downloaded from the NASA Level-1 and Atmosphere Archive & Distribution System (LAADS) [32] website. A modified gridding approach was used in this study, where the flux tower was located in the center of related 500 m or 1 km grids [33]. MODIS L1B radiance data from each swath were then gridded at 500 m or 1 km resolution for MODIS bands 1–2 with appropriate area weights of each MODIS observation. MODIS data were processed by the modified gridding method and the gridded observations were atmospherically corrected by the Multi-Angle Implementation of Atmospheric Correction (MAIAC) algorithm [34].

#### 2.2.3. BARC – Surface reflectance at USDA site with derivative analysis

To study the stability of reflectance for differing types of vegetation targets, Hyperion data acquired for the USDA BARC in Maryland were collected and used. The Hyperion Level 1T data were acquired in the summer and fall from 2005 – 2015 and calibrated to surface reflectance by the Fast Line-of-sight Atmospheric Analysis of Spectral Hypercubes (FLAASH) software [35] using default model parameters for typical summer/fall clear-sky atmospheric conditions. The acquisition time, sensor look angle, center location of the data and a visibility of 40 km were used as inputs for MODerate resolution atmospheric TRANsmission (MODTRAN) parameters.

Derivative analysis is a powerful tool that enhances the interpretation of data. Derivatives of second order or higher should be relatively insensitive to variations in illumination intensity whether caused by changes in sun angle, cloud cover, or topography [36]. Kalluri et al. [37] used spectral derivatives and single or multiple classifiers in land classification and achieved overall classification accuracy (expressed in percentage) that was significantly greater than was achieved when exploiting only the reflectance information. In a study by Ye et al. [38], the classification results in the spectral as well as derivative domains were fused by a logarithmic-opinion-pool rule and the results demonstrated that the algorithms improved classification accuracy even in cases with small training sample sizes. When combined with time-series analysis, derivative analysis reveals whether the signal is consistent, regardless of seasonal factors.

Derivatives are very sensitive to noise, so smoothing or minimizing random noise is important. Tsai and Philpot [36] examined several methods for smoothing of hyperspectral data. In this study the local polynomial regression fitting [39] was used to smooth the reflectance data before derivatives were calculated. 1^st^, 2^nd^, and 3^rd^ derivatives were calculated on the Hyperion spectra (Equation 2), considering the full width at half maximum (FWHM) value of each wavelength interval.

(2) $$\frac{\Delta r_{i}}{\Delta \lambda }=\frac{r_{i}-r_{i-1}}{{\left(\mathrm{FWHM}\right)}_{i}}$$

Where Δr_i_/Δλ is the derivative and Δr_i_ is the change in reflectance, Δλ is the change of wavelengths, and FWHM is the full width at half maximum for that wavelength

Various targets (each having 10 to 30 pixels) were identified from Hyperion data using the Region of Interest (ROI) tool of ENVI [40]. The mean reflectance of the targets were calculated for 152 calibrated bands of the Hyperion data. The calibrated bands consists of 5 pieces: 427 nm – 925 nm; 973 nm – 1114 nm; 1175 nm – 1326 nm; 1497 nm – 1790 nm and 2032 nm – 2355 nm. The width for the local filtering was 30 nm.

#### 2.2.4. Rail Road Valley Playa (RRVP) PICS – Surface Reflectance at a desert site

RRVP is among the PICSs endorsed by the Committee on Earth Observing Satellites (CEOS) to serve as a standard reference for the post-launch calibration of space-based optical imaging sensors [41]. RRVP is located in a large, dry lakebed in central Nevada and has a dry climate, typical of the high desert of the western USA [42]. The site is characterized by high reflectance, relatively high spatial and temporal uniformity, high midday sun elevation, and minimal cloud cover. The surface layers and composition are relatively smooth and spatially homogeneous, consisting of compacted clay-rich lacustrine deposits [43]. More description of the site and detailed spectral evaluations are available in Scott et al. [42], Teillet et al. [43], and Czapla-Myers et al. [44,45]. Because of its large size, RRVP is used for sensors with larger footprints (1–10 km), and is automated with instrumentation used extensively for the vicarious calibration of terrestrial imaging sensors covering the VNIR and SWIR wavelength ranges [44,45].

The Hyperion time series collection at RRVP was processed following the procedures outlined in Campbell et al. [46]. Hyperion TOA radiances were converted to surface reflectance using the Atmospheric CORrection Now (ACORN) software [47] and the module designed for pushbroom imaging spectrometers with cross-track spectral calibration variation. According to the date of acquisition, apparent surface reflectance was derived using either mid-latitude summer or winter atmospheric models. To preserve the original spectral properties and variability, the images were not geographically or geometrically rectified. This was valid as a prior study established that at RRVP, Moran I statistics vary between 0.81 and 0.95 across Hyperion’s spectral range (1 = strong positive spatial autocorrelation, 0 = spatially uncorrelated data), which was attributed to variation in soil moisture affects and differences in the mineral composition of the surface [46]. We used band subsetting to remove uncalibrated and overlapping bands, and bands adjacent to water absorption features, resulting in subsets having 171 bands (Table 4).

**Figure 4:**
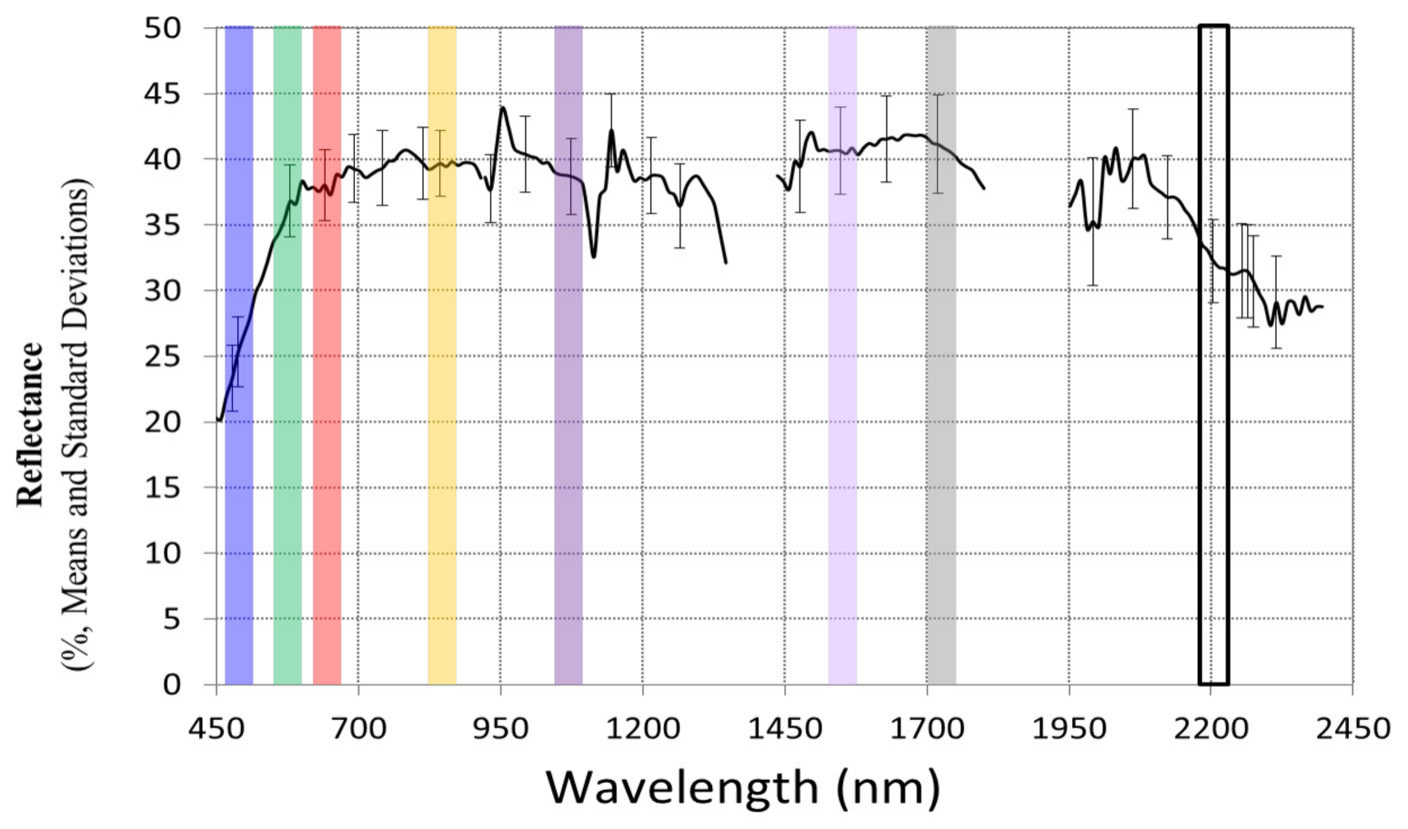
Mean reflectance and standard deviation for select wavelengths for the Rail Road Valley Playa (RRVP) site before late mission precession changes (2001–2008 data, n=15 images, ~10:05 am mean local time acquisition).

Time series of Hyperion data were used to determine spectral stability during the period previous to the precession change beginning in 2011 or during the changing satellite precession. Thirty-seven radiometrically corrected Level 1R images from 2001 – 2015 were converted to surface reflectance using the ACORN software. Mean reflectance and standard deviation (SD) were calculated for select wavelength bands (Figure 4). These wavelengths were chosen as representative of the spectral properties throughout the VNIR and SWIR wavelengths for RRVP. The mean was calculated with data from 2001 – 2008. Twenty-three pre-precessional and fourteen post-precessional images (see Figure 1 for dates) were used for the statistics of the change as delta reflectance (Δp).

#### 2.2.5. Libya-4 PICS – Hyperion time-series using different atmopheric correction models

To test differences of various atmospheric correction techniques and potential impacts of using Hyperion for cross calibration, we used a time-series of surface reflectance data from 2004 – 2016 in the Libya-4 desert PIC site, which is commonly used as a calibration site for Earth observing sensors.

Thirty-six images from WRS path 181, row 40 were co-registered. All data were nadir ±10 degrees and collected between May and September to reduce seasonal SZA effects. These data were atmospherically corrected to surface reflectance using ATREM, ACORN, and FLAASH [31,35,48]. ATREM uses a radiation transport model based on 6S, whereas ACORN and FLAASH use a more complex radiation transport model that retrieves atmospheric properties from bands near absorption features [49,50]. All three models account for differences in the measured upwelling radiance from differences in solar irradiance due to different acquisition dates and times, and all models had similar parameters applied for the Libya-4 PICS. Combined atmospheric model uncertainty was estimated using a quadrature statistic [51], expressed as the square root of the coefficient of variation of the sum of squares from each atmospheric correction approach.

A digital terrain model was also generated for this site using same date cross-track 50cm panchromatic WorldView-1 and WorldView-2 stereo imagery to characterize terrain slope impacts to reflectance products. Greater than 50 tie points were used and the RMSE for the product was less than 3.5m with a resulting resolution of 2m. This was done because we wanted to see if SZA change from precession combined with large dune shadowing at the site effected the results. More information about how these data were processed is available in [52].

## 3. Results

### 3.1. Park Falls, Wisconsin: EO-1 ALI NDVI vs. Landsat NDVI

**Figure 5:**
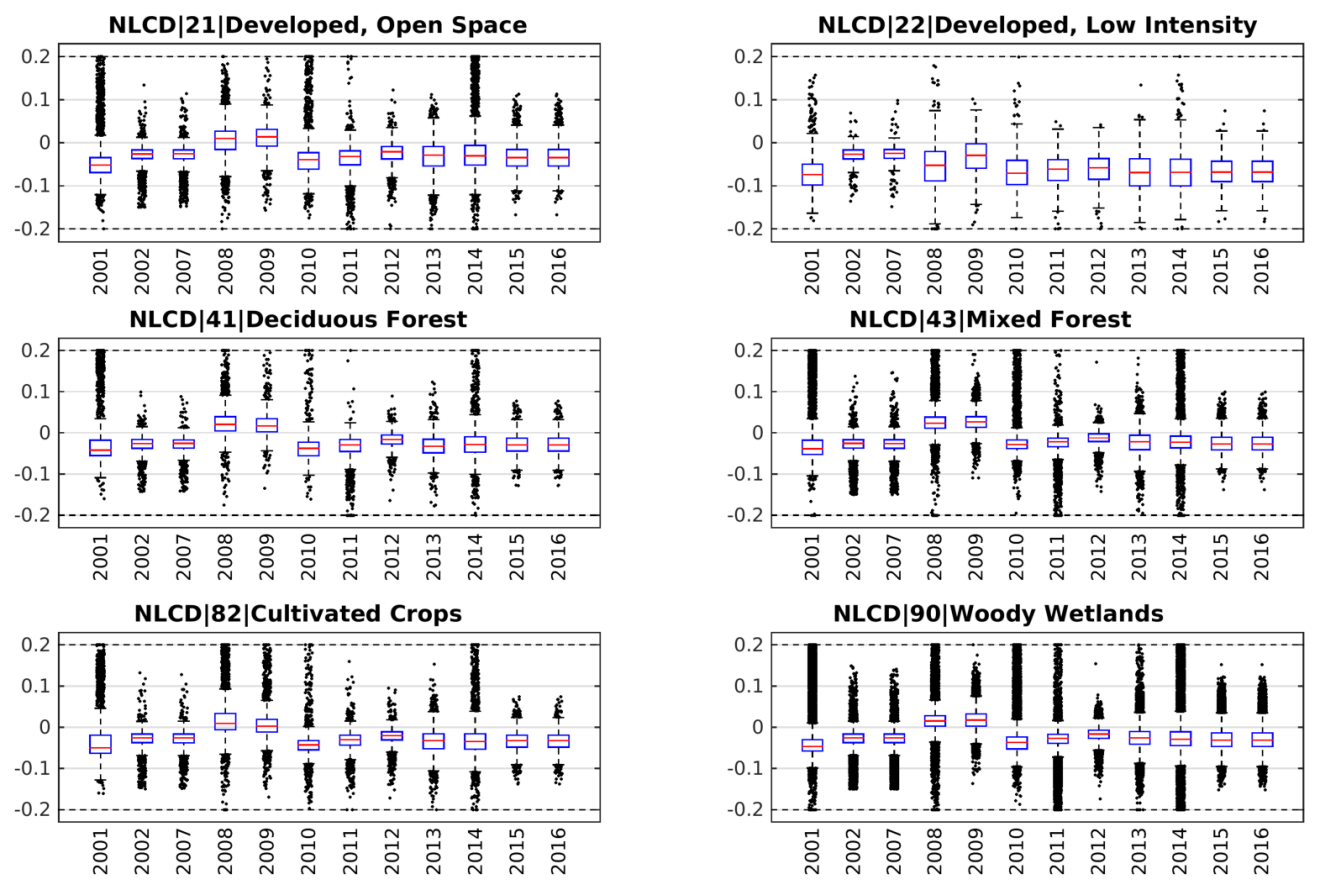
Statistics for median NDVI difference between Landsat and ALI sensors, per six National Land Cover Database (NLCD) classes. Red line is median, blue box is quartiles, and black crosses are outliers. NDVI values were derived from Top of Atmosphere (TOA) reflectance.

ALI analysis was comprised of two parts. First, we wanted to know the spectral difference between the calculated NDVI from the two sensors over the time series. In the comparison of NDVI from ALI and Landsat, the stratified images by cover type using the NLCD, only the low intensity development class (class 22) showed much variance, which had larger NDVI differences (between 0.05 and 0.10 Δ NDVI) than the others (see Figure 5). This difference may be attributed to a smaller sample size compared to the other land cover classes. Second, we wanted to identify changes in median ΔNDVI over time that occurred, possibly showing the manifestation of increasing differences in SZA due to orbital drift of EO-1. However, we show that there was no systematic trend in ΔNDVI occurring since the onset of precession. In Fig. 5 for all classes, the median difference hovers above and below zero with no observable trend. We hypothesized that if precession was affecting ALI data quality that the differences would increase in either a positive or negative fashion over time. Not including the low intensity development class (class 22), the highest deviation was 0.05 NDVI, found in several classes and years, but all before precession started in 2011.

### 3.2. Howland Forest, Maine – Hyperion NDVI comparison to MODIS NDVI

**Figure 6:**
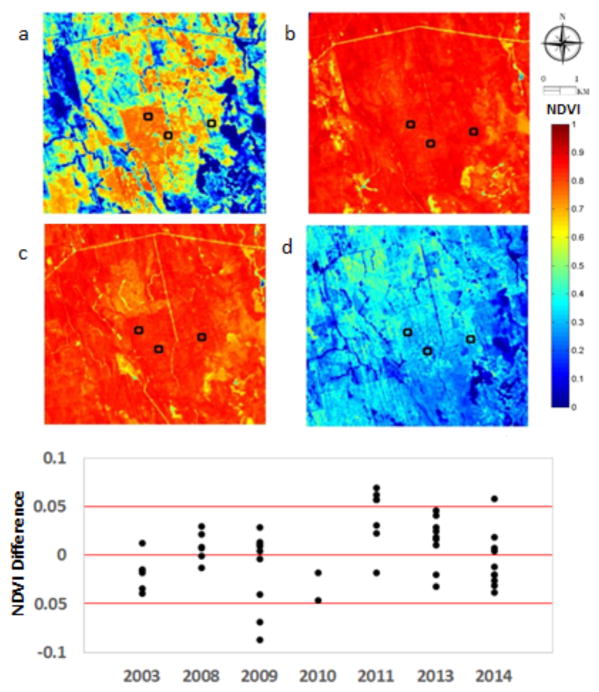
Upper chart: NDVI maps from EO-1 Hyperion images over the Howland Forest area in Maine across four seasons from: a) Spring; b) Summer; c) Fall; d) Winter. Three flux tower sites are indicated with black squares. Lower graph: NDVI difference between Hyperion and MODIS for the three flux tower sites in the Howland forest area of Maine from 2003 to 2014.

Phenological changes in NDVI were compared between EO-1 Hyperion and MODIS surface reflectances at three flux tower sites (US-Ho1, US-Ho2 and US-Ho3) in the Howland Experimental Forest, Maine. NDVI maps were derived from EO-1 Hyperion images over the 6 km area surrounding the three Howland Forest flux tower sites across four seasons from spring (March 5, 2014), summer (Aug. 12, 2014), fall (Sept. 22, 2008) and winter (Dec. 9, 2010). Figure 6 shows the expected NDVI seasonality, with both the early and late year observations of low NDVI and high mid-season NDVI responding to the presence of green leaves and higher photosynthesis.

For the areas around the three flux tower sites, NDVI was differenced between Hyperion and MODIS data (Figure 6, lower plot). Similar to other results, the NDVI difference is small (i.e. ΔNDVI < 0.15) and does not increase over time although it varies across years. Most of the large NDVI differences (from 0.10 – 0.15) occur in US-Ho3 site (East Tower, Harvest Site), which is not as homogeneous as the other two flux tower sites.

### 3.3. BARC – Hyperion surface reflectance derivative analysis

**Figure 7:**
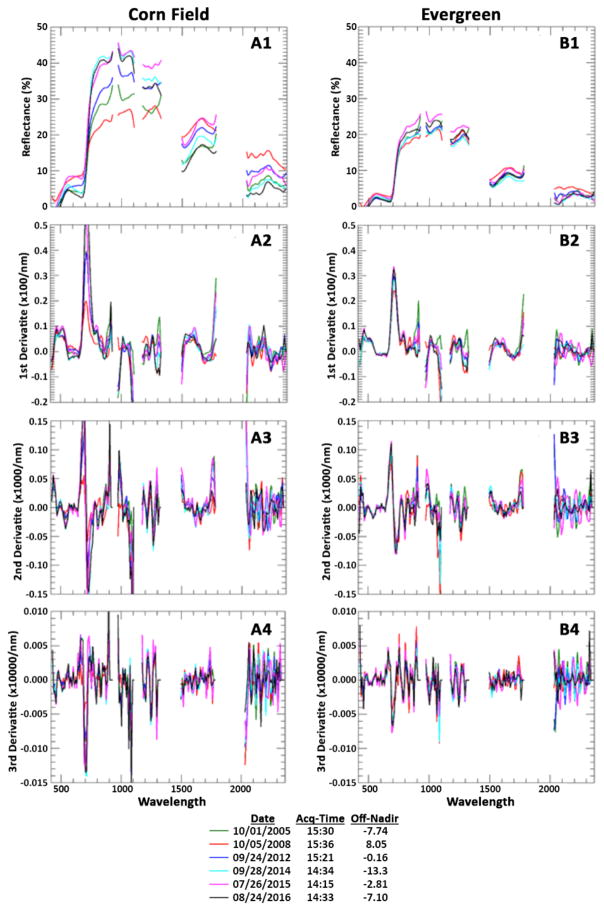
Spectrum (top row) and 1st, 2nd, 3rd derivatives (rows 2 to 4) of a Corn field (column A) and Evergreen patch (column B).

**Figure 8:**
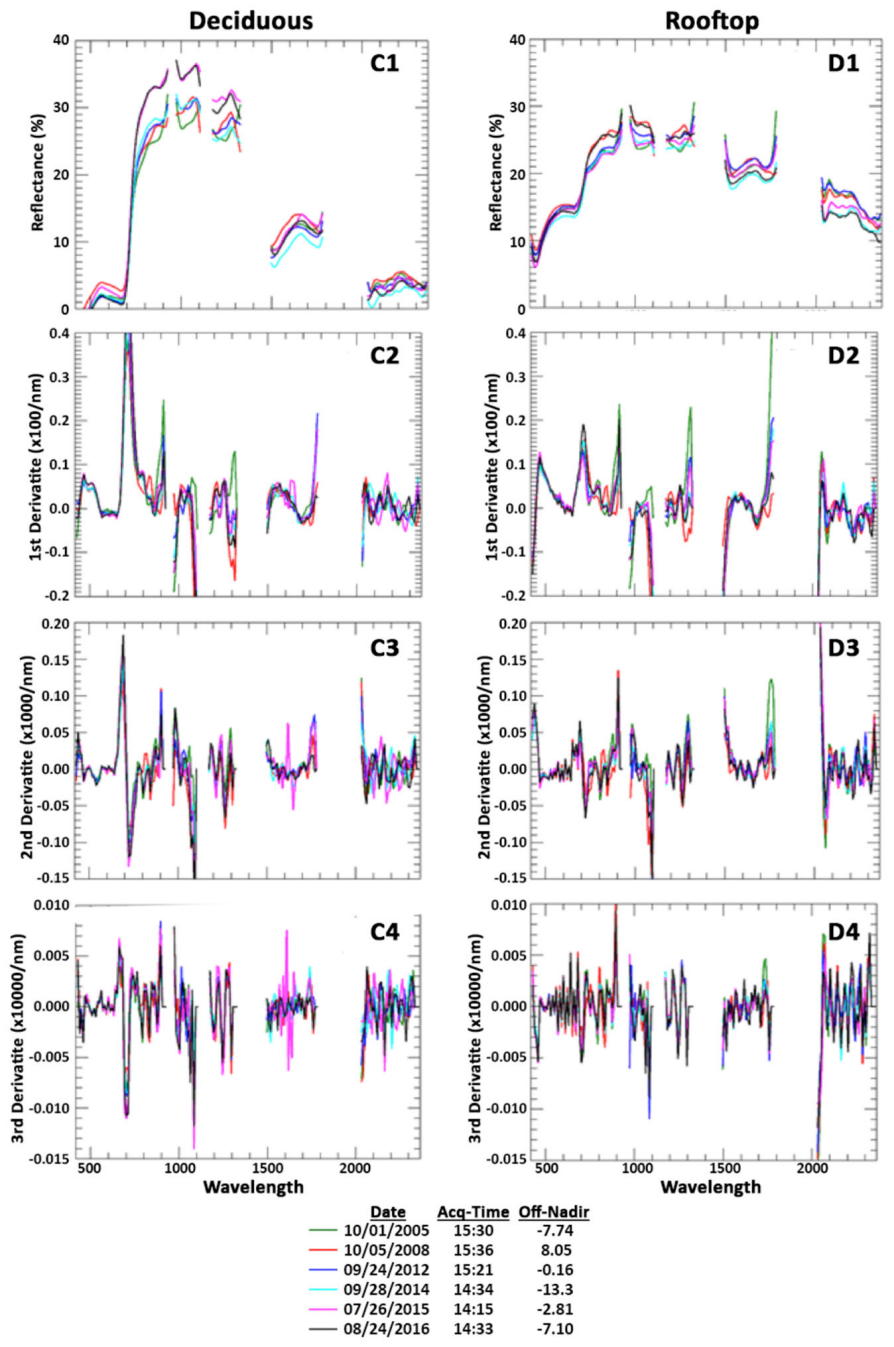
Spectrum (top row) and 1st, 2nd, 3rd derivatives (rows 2 to 4) of a deciduous patch (column C) and Rooftop (column D).

At this USDA site, we chose targets with varying degrees of seasonal differences to determine if derivative analysis displays a consistent signal throughout the Hyperion time-series. The spectral reflectance and derivatives from four targets (corn field, evergreen trees, deciduous trees, and top of building) are shown in Figure 7 & 8. As can be seen in the figures, the corn field has a very different spectral reflectance between the five dates due to the differences in planting and harvesting schedules in those years. The Evergreen patch and the top of a building, not surprisingly, have a much more uniform reflectance among dates. The derivatives show the consistency between different dates for a target and keep different features for different targets.

### 3.4. RRVP – Desert site surface reflectance time series

**Figure 9:**
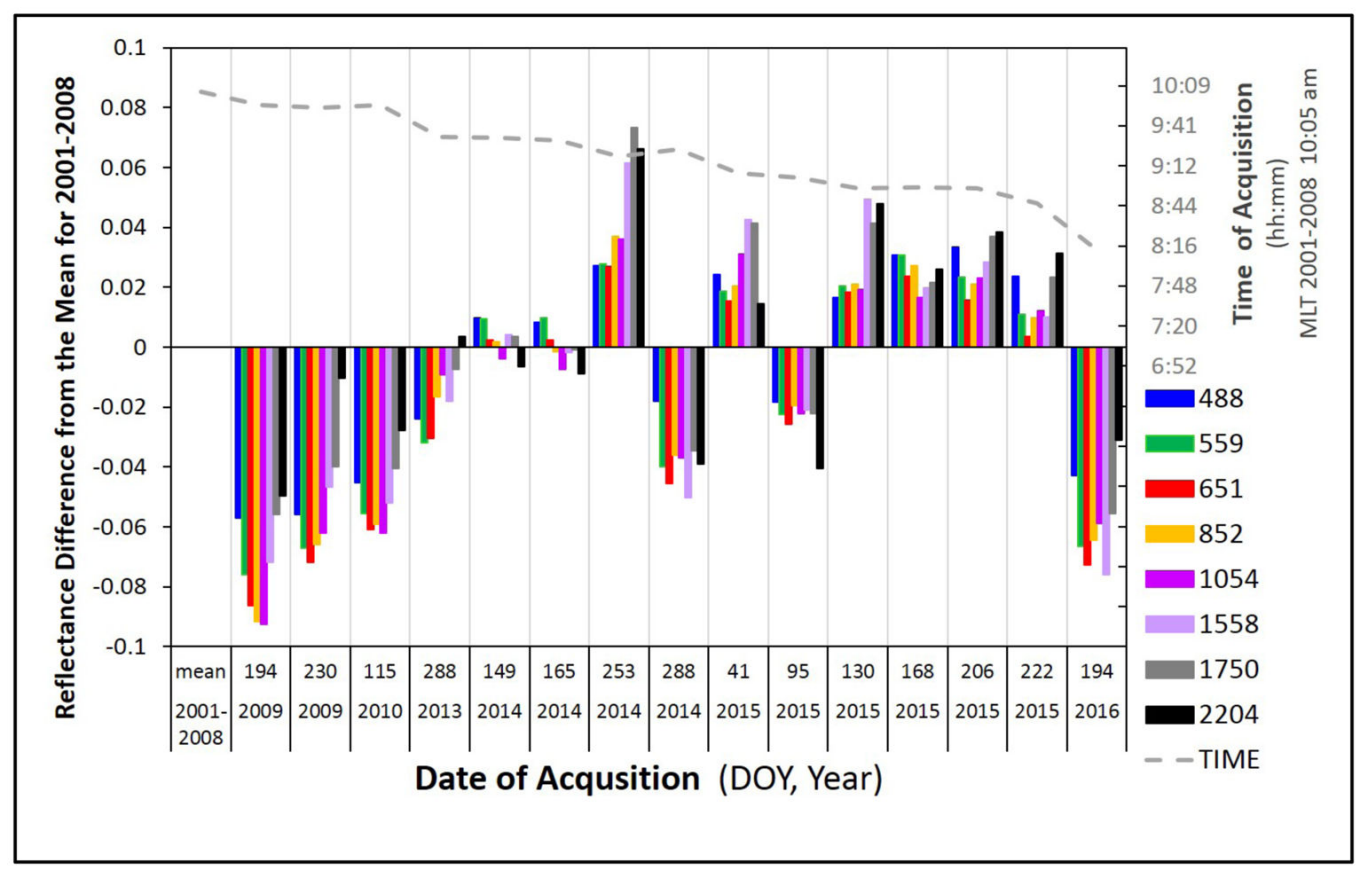
Change in reflectance anomaly (Δp) at select wavelengths. A mean was established by averaging values from 2001–2008. The dashed line above the bars shows the time of acquisitions and the key for wavelengths is presented in the lower right legend.

Similar to the ALI NDVI vs. Landsat NDVI study above, we wanted to look at deviations in reflectance of Hyperion data over time for a site with stable reflectance. Pre-precession means were calculated and differenced from subsequent years of acquisitions over the same target and wavelengths (Equation 3). In the first few years of precession, reflectance values are lower than pre-precession (Figure 9). From 2014 to early-2015 the values fluctuate about the mean and then from early-2015 Hyperion reflectance values increase to stay consistently above the pre-precession mean. However, the difference in reflectance continues to be within ± 5 – 9% of the mean prior to Δ precession. The regions of highest spectral stability (e.g, green, red edge, NIR) remain the same.

(3) $$(R_{i}=R_{\text{mean}\;2001-2008}-R_{\text{DOY},2009-2015}),$$

Where R_i_ is the differenced reflectance, R_mean_ is the calculated mean, and R_DOY_ is the reflectance per each observation after 2008.

### 3.5. Libya-4 PICS – Hyperion surface reflectance stability using three atmopheric correction models

**Figure 10:**
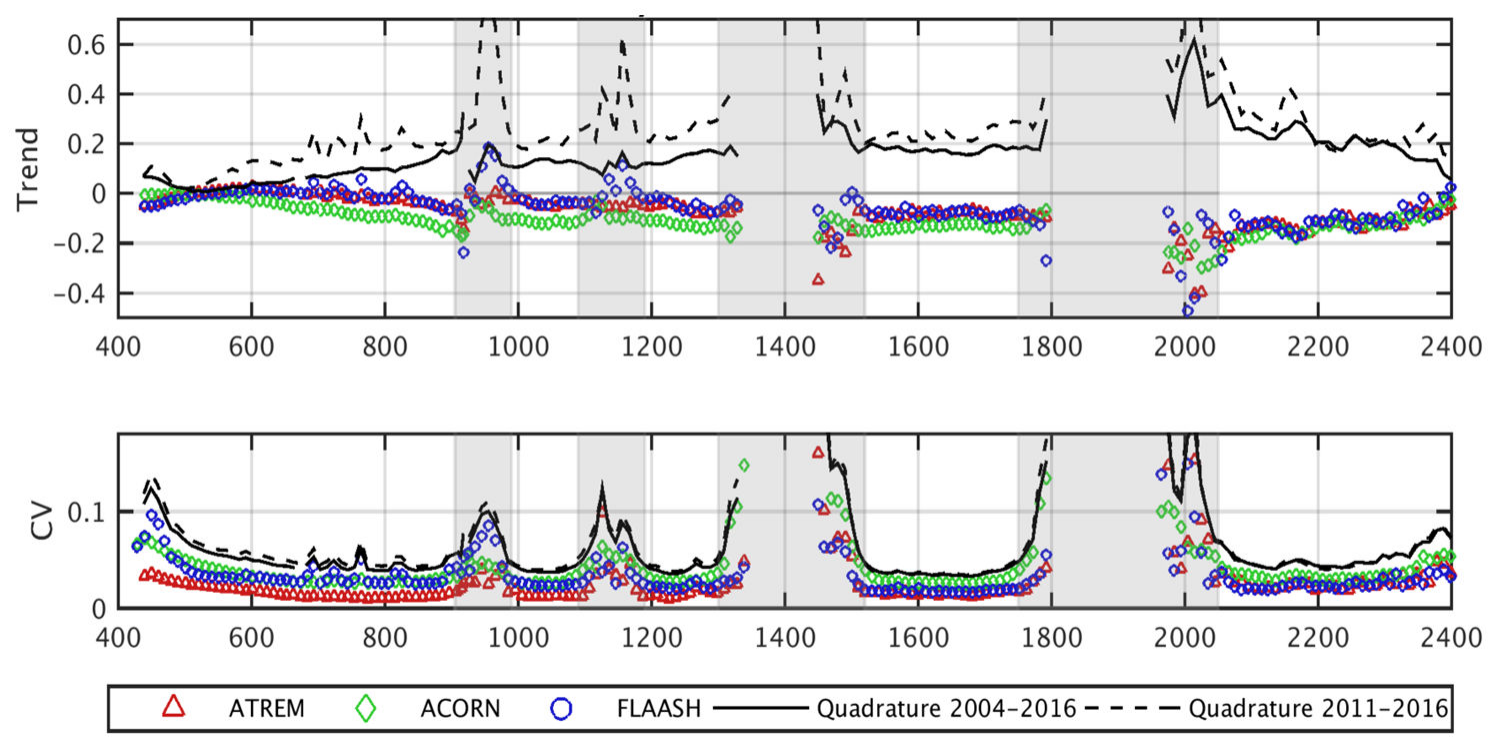
Means of 172 calibrated Hyperion bands from 36 images acquired from 2004 to 2016 after use of 3 atmospheric correction models: (top row) least squares fit linear trend in surface reflectance; (bottom row) coefficient of variation (CV). The quadrature (combined uncertainty) is calculated as the square root of the sum of squares. Gray areas indicate atmospheric absorption bands [52].

We studied Hyperion precession from 2004 through 2016 with three atmospheric correction algorithms to characterize temporal stability of surface reflectance products and to understand uncertainties introduced by terrain shadow in the Libya-PICS. Hyperion data were stable in most bands over this time period, independent of the atmospheric correction model used. However, the imagery degraded at different rates throughout the spectrum for the visible (VIS), NIR, infrared (IR), and SWIR between 2004 to 2016, as indicated through the several atmospheric correction techniques. The combined model variation expressed as a quadrature was within 10% for the VNIR and in most SWIR bands the variability was within 20%, excluding bands near atmospheric absorption features (Figure 10). The ATREM corrected images expressed the lowest change over time for the VNIR and IR, as described by the trend values (close to zero) and lowest CV. Overall, the CV was < 5% across the spectrum, except in bands adjacent to atmospheric absorption regions. No significant (p < 0.01) or rapid degradation was apparent for any spectral interval during the precession period for the three correction approaches. The quadrature values were similar for the entire dataset (Quadratures 2004–2016) as well as for the last five years of the mission (Quadratures 2011–2016). We found that locations having terrain slopes greater than 15° introduced a peak value in anomaly trends as compared to dune flats of 0 to 10°. Trends progressively increased for slopes up to 15°, then oscillated 2 to 5% in the VIS and > 20% in the SWIR, due to Hyperion imaging both the illuminated and shaded portions of dunes. The BRDF information about large dunes in the Libya-4 PICS is important for cross-calibrating Earth observing sensors.

## 4. Discussion

The catalyst for this study was EO-1’s declining orbit and this work evaluated how it affected its instrument’s data quality and stability though time. In general, we demonstrate that surface reflectance retrievals were not seriously affected during the late mission precession period, typically being within 5% for VIS and 10% longer wavelengths for most surfaces, although the Libya-4 PICS desert variation was slightly higher due mostly, we suspect, to large dunes casting larger shadows from increased SZA from earlier overpass times. Other land cover types, including croplands and deciduous forests, were shown to have differences in reflectance between dates, but showed consistency when looking at their derivatives. All land cover types that we looked at, however, did not show marked increase or decrease in NDVI over the full precession range of our time series. It should also be stated that we avoided high off-nadir sensor view angles when selecting our images to minimize variance due to view geometry differences.

The effect of precession on other sensors has been studied and we can compare our results to theirs. Swinnen, et al.[22] measured orbital drift and its effects on Satellite Pour l’Observation de la Terre (SPOT) data, finding somewhat similar or slightly higher differences in spectral reflectance that ranged from 10% – 20% when in reference to another sensor, but the impact on NDVI was negligible. We assume that the reason their study obtained slightly higher variances than ours was because the two SPOT sensors that were compared (i.e., VGT1 and VGT2) had differences in calibration accuracies, as well as slightly different spectral response functions

This work provides insight on the limits of increasing SZAs on EO-1 surface reflectance data quality. This issue is important not only for the creation of consistent long-term satellite time series, but also help to define issues in combining data from multiple satellites with different overpass times, as have been proposed for future satellite constellations, and to merge existing or past data collections. Atmospheric correction models account for differences in the measured upwelling radiance for different acquisition dates and times and results could differ if only TOA products are used. Future work could study the limits of even earlier crossing times to see at what point the increased SZA starts to negatively affect data quality. Hagolle [53] found that the relative difference in reflectance values and NDVI increased moderately with increase in initial SZA up to 50°, but beyond this, an increase in SZA resulted in an exponential change in relative difference. While this result is important, it was based on simulated SPOT data and only evaluated four multispectral bands. Our study is current as of late-2016, when the minimum SZA reached 50° (see Fig. 3). Early in 2017, the last images were collected and the mission was decommissioned. At this point the minimum SZA was around 55°, preventing us from determining the effects on data as the sun approaches the horizon at overpass time.

Nevertheless, the first space-based measurements of a large methane leak from the Aliso Canyon, California super emitter were captured by Hyperion in January 2016 under low winter sun angles, and later verified by aircraft observations, demonstrating that even a nearly invisible gas plume above a complex landscape could be detected from orbit with a spectrometer [54]. This opens new possibilities for future monitoring capabilities if NASA chooses to support a mission similar to the one identified by the National Research Council in the 2007 Decadal Survey for Earth Sciences, which defined a Pre-Phase-A mission, the Hyperspectral InfraRed Imager (HyspIRI).

## 5. Conclusions

In this study, multispectral and hyperspectral remote sensing imagery were evaluated from the EO-1’s two spectral imagers, ALI and Hyperion, in comparison with data from the Landsat and MODIS sensors. Our analyses were done at a mid-latitude mixed forest site, two desert PIC sites, a USDA agricultural research site, and a northern experimental forest. ALI NDVI was compared to Landsat NDVI, Hyperion reflectance was examined before and during orbital precession using time-series for Hyperion NDVI data compared to MODIS NDVI and using spectral derivative analyses. Additionally, we evaluated the role that different atmospheric correction algorithms had on time-series Hyperon imagery. We have shown in this study that the sensors onboard the EO-1 satellite have produced robust products for scientific analysis of the Earth during its entire 16 year mission. This study took a multi-faceted approach to quantify EO-1 data quality and we determined that no marked decline exists for either ALI or Hyperion when compared with other highly calibrated and stable sensors in a diverse set of locations. The variability is typically within 5% for the VNIR and within 10% for the SWIR wavelengths, excluding bands near atmospheric absorption features, which is in the range of previous EO-1 data quality estimates that were made before the satellite’s orbit started precession [3,12]. Lastly, it was found that this variability in retrieved surface reflectance is not seriously affected among three commonly employed atmospheric correction techniques. This is encouraging for time-series analysis, when it is often pertinent to correct for differing atmospheric conditions that may be present.

It is important to note that these results are current as of mid-2016. Median overpass times will continue to get earlier and greater solar zenith angles will be experienced. In October 2016 the overpass time reached 8AM local time and the satellite started the decommission protocol, which will be completed in March 2017. In between that time users should be cautious when using EO-1 data as data quality under these circumstances has not been evaluated.

## Figures and Tables

**Table 1:** Dominant land cover types and Northern Hemisphere study site locations used in analyses

Site Name	Central Coordinates (longitude, latitude)	Dominant Land Cover
Park Falls, Wisconsin	90.18° W, 45.58° N	Mixed hardwood forest
Howland Forest, Maine	68.5° W, 45.21° N	Mixed coniferous forest
BARC^1^, Maryland	76.85° W, 39.03° N	Evergreen, Corn Field
RRVP^2^, Nevada PICS	115.69° W, 38.5° N	Desert
Libya- 4 PICS^3^	24.40° E, 28.53° N	Desert

1 Beltsville Agricultural Research Center,

2 Rail Road Valley Playa,

3 Pseudo-Invariant Calibration Site

**Table 2:** Coupled image pairs used for the Park Falls, WI analysis.

Landsat date	ALI date	Off nadir
8/17/2016	8/17/2016	20.10
8/15/2015	8/13/2015	−0.30
8/12/2014	8/09/2014	−5.70
9/26/2013	9/27/2013	6.10
8/06/2012	8/05/2012	4.50
8/20/2011	8/27/2011	−2.72
8/17/2010	8/17/2010	−2.50
9/23/2009	9/21/2009	−1.00
9/12/2008	9/09/2008	5.60
6/30/2007	6/30/2007	−7.20
8/11/2002	8/11/2002	3.50
5/04/2001	5/04/2001	0.13

**Table 3:** Wavelengths ranges per band for each ALI, TM, and ETM+ sensor

	EO-1 ALI	Landsat 5	Landsat 7
	Wavelength (μm)	Wavelength (μm)	Wavelength (μm)
Pan 1	0.48 – 0.69		.52–.90
Blue′	0.43 – 0.45		
Blue	0.45 – 0.52	0.45–0.52	0.45–0.52
Green	0.53 – 0.61	0.52–0.60	0.52–0.60
Red	0.63 – 0.69	0.63–0.69	0.63–0.69
NIR	0.78 – 0.81	0.76–0.90	0.77–0.90
NIR′	0.85 – 0.89		
SWIR′	1.20 – 1.30		
SWIR 1	1.55 – 1.75	1.55–1.75	1.55–1.75
TIR		10.40–12.50	10.40–12.50
SWIR 2	2.08 – 2.35	2.08–2.35	2.09–2.35

1 Pan = Pancromatic

**Table 4:** Subset of bands used in RRVP study

Array	Bands	Wavelengths (nm)
VNIR	8 – 57 (49)	426 – 926
SWIR1	7 – 120 (41)	993 – 1346
SWIR2	129 – 165 (36)	1518 – 1800
SWIR3	179 – 224 (5)	1942 – 2395

Total	(171)	

## Abbreviations

The following abbreviations are used in this manuscript

ACORN: Atmospheric CORrection Now
ALI: Advanced Land Imager
ATREM: Atmosphere Removal
BARC: Beltsville Agricultural Research Center
BRDF: Bi-Directional Reflectance Distribution Function
CEOS: Committee on Earth Observing Satellites
CV: Coefficient of Variation
EO-1: Earth Observing One
ETM+: Enhanced Thematic Mapper plus
FLAASH: Fast Line-of-sight Atmospheric Analysis of Spectral Hypercubes
TM: Thematic Mapper
MODIS: Moderate Resolution Imaging Spectroradiometer
NDVI: Normalized Difference Vegetation Index
NIR: Near Infrared
PICS: Pseudo-Invariant Calibration Site
RRVP: Rail Road Valley Playa
SNR: Signal to Noise Ratio
SWIR: Short Wave Infrared
SZA: Solar Zenith Angle
TOA: Top of Atmosphere
USGS: United States Geological Survey
VNIR: Visible Near Infrared
WRS: Worldwide Reference System

## References

[1] Elmore AJ, Mustard JF (2003). Precision and Accuracy of EO-1 Advanced Land Imager (ALI) Data for Semiarid Vegetation Studies. Ieee Transactions on Geoscience and Remote Sensing.

[2] Datt B, McVicar TR, Van Niel TG, Jupp DLB, Pearlman JS (2003). Preprocessing EO-1 Hyperion Hyperspectral Data to Support the Application of Agricultural Indexes. Ieee Transactions on Geoscience and Remote Sensing.

[3] Ungar SG, Pearlman JS, Mendenhall JA, Reuter D (2003). Overview of the Earth Observing One (EO-1) Mission. Ieee Transactions on Geoscience and Remote Sensing.

[4] Bryant R, Moran MS, McElroy SA, Holifield C, Thome KJ, Miura T, Biggar SF (2003). Data Continuity of Earth Observing 1 (EO-1) Advanced Land Imager (ALI) and Landsat Tm and ETM. Ieee Transactions on Geoscience and Remote Sensing.

[5] Pu R, Yu Q, Gong P, Biging GS (2005). EO-1 Hyperion, ALI and Landsat 7 ETM+ Data Comparison for Estimating Forest Crown Closure and Leaf Area Index. International Journal of Remote Sensing.

[6] Roy DP, Wulder MA, Loveland TR, Woodcock CE, Allen RG, Anderson MC, Helder D, Irons JR, Johnson DM, Kennedy R, Scambos T, Schaaf CB, Schott JR, Sheng Y, Vermote EF, Belward AS, Bindschadler R, Cohen WB, Gao F, Hipple JD, Hostert P, Huntington J, Justice CO, Kilic A, Kovalskyy V, Lee ZP, Lymbumer L, Masek JG, McCorkel J, Shuai Y, Trezza R, Vogelmann J, Wynne RH, Zhu Z (2014). Landsat-8: Science and Product Vision for Terrestrial Global Change Research. Remote Sensing of Environment.

[7] Irons JR, Dwyer JL, Barsi JA (2012). The Next Landsat Satellite: The Landsat Data Continuity Mission. Remote Sensing of Environment.

[8] Ungar S (2016). Personal Communication.

[9] Middleton EM, Ungar SG, Mandl DJ, Ong L, Frye SW, Campbell PE, Landis DR, Young JP, Pollack NH (2013). The Earth Observing One (EO-1) Satellite Mission: Over a Decade in Space. Ieee Journal of Selected Topics in Applied Earth Observations and Remote Sensing.

[10] Fagan ME, DeFries RS, Sesnie SE, Arroyo-Mora JP, Soto C, Singh A, Townsend PA, Chazdon RL (2015). Mapping Species Composition of Forests and Tree Plantations in Northeastern Costa Rica with an Integration of Hyperspectral and Multitemporal Landsat Imagery. Remote Sensing.

[11] White JC, Gomez C, Wulder MA, Coops NC (2010). Characterizing Temperate Forest Structural and Spectral Diversity with Hyperion EO-1 Data. Remote Sensing of Environment.

[12] Blanco PD, del Valle HF, Bouza PJ, Metternicht GI, Hardtke LA (2014). Ecological Site Classification of Semiarid Rangelands: Synergistic Use of Landsat and Hyperion Imagery. Int J Appl Earth Obs.

[13] Chen S, Fang L, Li H, Chen W, Huang W (2011). Evaluation of a Three-Band Model for Estimating Chlorophyll-a Concentration in Tidal Reaches of the Pearl River Estuary, China. ISPRS-J Photogramm Remote Sens.

[14] Chen S, Fang L, Li H, Chen W, Huang W (2011). Evaluation of a Three-Band Model for Estimating Chlorophyll-a Concentration in Tidal Reaches of the Pearl River Estuary China. ISPRS Journal of Photogrammetry and Remote Sensing.

[15] Brando VE, Dekker AG (2003). Satellite Hyperspectral Remote Sensing for Estimating Estuarine and Coastal Water Quality. Ieee Transactions on Geoscience and Remote Sensing.

[16] Chudnovsky A, Kostinski A, Herrmann L, Koren I, Nutesku G, Ben-Dor E (2011). Hyperspectral Spaceborne Imaging of Dust-Laden Flows: Anatomy of Saharan Dust Storm from the Bodele Depression. Remote Sensing of Environment.

[17] Chien S, Doubleday J, Mclaren D, Tran D, Tanpipat V, Chitradon R, Boonya-aroonnet S, Thanapakpawin P, Mandl D (2013). Monitoring Flooding in Thailand Using Earth Observing One in a Sensorweb. Ieee Journal of Selected Topics in Applied Earth Observations and Remote Sensing.

[18] Davies AG, Chien S, Doubleday J, Tran D, Thordarson T, Gudmundsson MT, Hoskuldsson A, Jakobsdottir SS, Wright R, Mandl D (2013). Observing Iceland’s Eyjafjallajokull 2010 Eruptions with the Autonomous Nasa Volcano Sensor Web. J Geophys Res-Sol Ea.

[19] Mandl D, Frye S, Cappelaere P, Handy M, Policelli F, Katjizeu M, Van Langenhove G, Aube G, Saulnier JF, Sohlberg R, Silva JA, Kussul N, Skakun S, Ungar SG, Grossman R, Szarzynski J (2013). Use of the Earth Observing One (EO-1) Satellite for the Namibia Sensorweb Flood Early Warning Pilot. Ieee Journal of Selected Topics in Applied Earth Observations and Remote Sensing.

[20] Davies AG, Chien S, Baker V, Doggett T, Dohm J, Greeley R, Ip F, Castano R, Cichy B, Rabideau G, Tran D, Sherwood R (2006). Monitoring Active Volcanism with the Autonomous Sciencecraft Experiment on EO-1. Remote Sensing of Environment.

[21] Ramsey MS, Flynn LP (2004). Strategies, Insights, and the Recent Advances in Volcanic Monitoring and Mapping with Data from Nasxs Earth Observing System. J Volcanol Geoth Res.

[22] Swinnen E, Verbeiren S, Deronde B, Henry P (2014). Assessment of the Impact of the Orbital Drift of SPOT-VGT1 by Comparison with SPOT-VGT2 Data. International Journal of Remote Sensing.

[23] Devasthale A, Grassl H (2007). Dependence of Frequency of Convective Cloud Occurrence on the Orbital Drift of Satellites. International Journal of Remote Sensing.

[24] Negri AJ, Bell TL, Xu LM (2002). Sampling of the Diurnal Cycle of Precipitation Using Trmm. J Atmos Ocean Tech.

[25] USGS EROS www.Earthexplorer.Com.

[26] Rouse JW, Haas RH, Schell JA, Deering DW (1974). Monitoring Vegetation Systems in the Great Plains with Erts. NASA Special Publication 351.

[27] Tucker CJ (1979). Red and Photographic Infrared Linear Combinations for Monitoring Vegetation. Remote Sensing of Environment.

[28] Galvao LS, Ponzoni FJ, Epiphanio JCN, Rudorff BFT, Formaggio AR (2004). Sun and View Angle Effects on Ndvi Determination of Land Cover Types in the Brazilian Amazon Region with Hyperspectral Data. International Journal of Remote Sensing.

[29] Homer C, Dewitz J, Yang LM, Jin S, Danielson P, Xian G, Coulston J, Herold N, Wickham J, Megown K (2015). Completion of the 2011 National Land Cover Database for the Conterminous United States - Representing a Decade of Land Cover Change Information. Photogrammetric Engineering and Remote Sensing.

[30] Gao BC, Davis CO (1997). Development of a Line-by-Line-Based Atmosphere Removal Algorithm for Airborne and Spaceborne Imaging Spectrometers. P Soc Photo-Opt Ins.

[31] Gao BC, Heidebrecht KB, Goetz AFH (1993). Derivation of Scaled Surface Reflectances from Aviris Data. Remote Sensing of Environment.

[32] NASA Level-1 and Atmosphere Archive & Distribution System (Laads).

[33] Zhang QY, Cheng YB, Lyapustin AI, Wang YJ, Gao F, Suyker A, Verma S, Middleton EM (2014). Estimation of Crop Gross Primary Production (GPP): fAPAR(Chl) Versus MOD15A2 FPAR. Remote Sensing of Environment.

[34] Lyapustin AI, Wang YJ, Laszlo I, Hilker T, Hall FG, Sellers PJ, Tucker CJ, Korkin SV (2012). Multi-Angle Implementation of Atmospheric Correction for MODIS (MAIAC): 3. Atmospheric Correction Remote Sensing of Environment.

[35] Felde GW, Anderson GP, Cooley TW, Mathew MW, adler-Golden SM, Berk A, Lee J Analysis of Hyperion Data with the FLAASH Atmospheric Correction Algorithm.

[36] Tsai F, Philpot W (1998). Derivative Analysis of Hyperspectral Data. Remote Sensing of Environment.

[37] Kalluri HR, Prasad S, Bruce LM (2010). Decision-Level Fusion of Spectral Reflectance and Derivative Information for Robust Hyperspectral Land Cover Classification. Ieee Transactions on Geoscience and Remote Sensing.

[38] Ye Z, He MY, Fowler JE, Du Q Hyperspectral Image Classification Based on Spectra Derivative Features and Locality Preserving Analysis.

[39] Cleveland WS (1979). Robust Locally Weighted Regression and Smoothing Scatterplots. J Am Stat Assoc.

[R40] 40Exelis Visual Information Solutions, B., Colorado.

[41] Teillet PM, Barsi JA, Chander G, Thome KJ, Xiong J (2007). Prime Candidate Earth Targets for the Post-Launch Radiometric Calibration of Space-Based Optical Imaging Instruments. Proceedings of SPIE.

[42] Scott KPTKJ, Brownlee MR (1996). Evaluation of the Railroad Valley Playa for Use in Vicarious Calibration. Proc of SPIE.

[43] Teillet PM, Ren XM (2008). Spectral Band Difference Effects on Vegetation Indices Derived from Multiple Satellite Sensor Data. Canadian Journal of Remote Sensing.

[44] Czapla-Myers JS, Thome KJ, Biggar SF (2008). Design, Calibration, and Characterization of a Field Radiometer Using Light-Emitting Diodes as Detectors. Applied Optics.

[45] Czapla-Myers J, Ong L, Thome K, McCorkel J (2016). Validation of EO-1 Hyperion and Advanced Land Imager Using the Radiometric Calibration Test Site at Railroad Valley, Nevada. Ieee Journal of Selected Topics in Applied Earth Observations and Remote Sensing.

[46] Campbell PKE, Middleton EM, Thome KJ, Kokaly RF, Huemmrich KF, Lagomasino D, Novick KA, Brunsell NA (2013). EO-1 Hyperion Reflectance Time Series at Calibration and Validation Sites: Stability and Sensitivity to Seasonal Dynamics. Ieee Journal of Selected Topics in Applied Earth Observations and Remote Sensing.

[47] AIG (2001). ACORN User’s Guide, Stand Alone Version. Analytical Imaging and Geophysics LLC.

[48] Kruse FA, Boardman JW, Huntington JF (2003). Comparison of Airborne Hyperspectral Data and EO-1 Hyperion for Mineral Mapping. Ieee T Geosci Remote.

[49] Gao BC, Goetz AFH (1990). Column Atmospheric Water Vapor and Vegetation Liquid Water Retrievals from Airborne Imaging Spectrometer Data. Journal of Geophysical Research.

[50] Kruse FA (2004). Comparison of ATREM, ACORN, and FLAASH Atmospheric Corrections Using Low-Altitude AVIRIS Data of Boulder, Colorado.

[51] Taylor JR (1939). An Introduction to Error Analysis the Study of Uncertainties in Physical Measurements.

[52] Neigh CSR, McCorkel J, Campbell P, Ong L, Ly V, Landis D, Fry S, Middleton EM (2016). Monitoring Orbital Precession of EO-1 Hyperion with Three Atmospheric Correction Models in the Libya-4 Pics. IEEE Geoscience and Remote Sensing Letters.

[53] Hagolle O (2007). Effet D’un Changement D’Heure De Passage Sur Les Series Temporelles De Donnees De L’Instrument Vegetation.

[54] Thompson DR, Thorpe AK, Frankenberg C, Green RO, Duren R, Guanter L, Hollstein A, Middleton E, Ong L, Ungar S (2016). Space-Based Remote Imaging Spectroscopy of the Aliso Canyon CH4 Superemitter. Geophysical Research Letters.

